# Global Publication Trends and Research Hotspots of Diabetes and Osteoporosis

**DOI:** 10.2174/0118715303325060241211094931

**Published:** 2025-01-14

**Authors:** Yuan Zhang, Qi Ren, Panmei Zhao, Qinghua Zou

**Affiliations:** 1 Department of Rheumatology and Immunology, The First Affiliated Hospital of Army Medical University, Chongqing, China

**Keywords:** Diabetes, osteoporosis, bibliometrics, citespace, visualization, global publication trends

## Abstract

**Background:**

Diabetes and osteoporosis, as chronic diseases with high incidence, have caused deep concern in the field of global public health due to their high morbidity and mortality. More importantly, the complex and close relationship between diabetes and osteoporosis has gradually become the focus of scientific research. It is very meaningful to carry out bibliometric analysis in the research field of diabetes and osteoporosis to describe the current international trend and present a visual representation of the past and emerging trends of diabetes and osteoporosis in the past decade.

**Methods:**

In this study, the characteristics of the articles on “diabetes and osteoporosis” retrieved and downloaded from the Web of Science Core Collection (WoSCC) database from January 1, 2011 to December 1, 2022 were analyzed by bibliometrics to clarify the evolution and theme trends between the two diseases. Citespace software was used for data analysis and visualization, including countries, academic institutions, journals, authors, subject categories, keywords, references, and citations. In addition, some important subtopics identified by bibliometric characterization were further discussed and reviewed.

**Results:**

Finally, 3372 articles were included in the analysis, including a total of 96 countries, 407 organizations, 1161 journals, and 617 keywords. Articles related to diabetes and osteoporosis were first published in 2011 and then showed an increasing trend year by year. The United States, China, Italy, England, and Japan were the top 5 countries associated with the largest number of publications. University of California-San Francisco, China Medical University, University of Toronto, Shanghai Jiao Tong University, and Mayo Clinic were the top 5 academic institutions in terms of the number of published papers. The top 5 authors with the highest number of publications were William D, Ann V, Nicola, Peter, and Toshitsugu. Osteoporosis International has published 130 articles in this field, ranking first among highly productive journals. In addition to diabetes and osteoporosis, the most frequently used keywords were bone mineral density, obesity, and fracture.

**Conclusion:**

More and more studies have been conducted on diabetes and osteoporosis, and the current research mainly focuses on the pathogenesis of various chronic diseases. In the future, more attention may be paid to the prevention and management of these two chronic diseases and the production and application of new drugs.

## 1 INTRODUCTION

In recent years, more and more pieces of evidence have shown that diabetes and osteoporosis are related diseases with significant associated morbidity and mortality [1]. Diabetes is one of the most common chronic diseases characterized by abnormal hyperglycemia resulting from defects in insulin secretion or insulin action, which is distributed all over the world, especially in developing countries [2]. According to the IDF Diabetes Atlas 10th edition [3], there are 537 million adults living with diabetes worldwide, and this figure is predicted to rise to 643 million by 2030 and 783 million by 2045. The prevalence rate of diabetes is positively correlated with age. The lowest prevalence rate is 2.2% among people aged 20-24, while people aged 75-79 are about 24.0%. With the aging of the global population, the proportion of diabetes patients over 60 years old will be higher and higher. Well-known persistent hyperglycemia of diabetes is associated with long-term complications, such as microvascular diseases and macrovascular diseases [4]. However, bone metabolism and bone mineralization may also be affected by diabetes, and diabetic bone disease, a new kind of complication of diabetes, has received more and more attention from endocrinologists [5].

Osteoporosis is a skeletal disorder characterized by reduced bone mass and altered bone quality, predisposing to an increased risk of fractures [6]. The pathogenesis of osteoporosis is very complex. Bone minerals, hormones, cytokines, bone absorption markers, bone formation markers, genes, receptors, trace elements, and other factors jointly regulate bone metabolism and homeostasis of bone environment, which is also an important factor determining bone quality and bone strength [7]. With the increase in the proportion of the global elderly population and the growth of life expectancy under the background of good medical resources, the population of osteoporosis is predicted to boom in the near future. It is reported that approximately 10 million Americans over the age of 50 suffer from osteoporosis, with a further 34 million at risk of the disease [8]. An epidemiological study from the UK shows that one in two women and one in five men aged over 50 years will be vulnerable to osteoporotic fractures in their lifetime [9]. As a country accounting for one-fifth of the global population and an even higher percentage of the elderly population, China also faces a severe burden of disease. It is estimated that nearly 8.2% of the elderly in China suffered from osteoporosis in 2010, which will increase to 13.6% in 2050 [10].

There is a well-established relationship between diabetes and bone disease that is less well-defined, but recent data seem to suggest that diabetes and the complications associated with it can be detrimental to bone health. Notably, patients with diabetes mellitus have an increased risk of osteoporosis-associated fractures [11-13]. In type 1 diabetes, impaired bone formation is a result of absolute deficiency of insulin and insulin-like growth factor-1 (IGF-1), which leads to the risk of osteoporosis being 6 times higher than that of the general population [14]. However, the occurrence of osteoporosis in T2DM is twice that of non-diabetic individuals. The potential pathophysiological mechanism is that hyperinsulinemia in patients can damage bone metabolism through IRS-1 and IRS-2 surface receptors on osteoblasts [15], and high levels of insulin can decrease the concentration of sex hormone binding globulin (SHBG), affecting the levels of estradiol and testosterone to reduce bone quality [16]. In addition, it appears that thiazolidinediones, medications used in the treatment of diabetes, can also cause bone loss and increase the risk of fracture [17, 18].

Although the articles on “diabetes and osteoporosis” have expanded considerably, as far as we know, there is no publication comprehensively summarizing the current status and predicting future trends in this field. Bibliometrics is a discipline that applies mathematics and statistical methods to analyze the characteristics and development trends qualitatively and quantitatively of the relevant research literature [19]. At present, this method has been widely used in hot spot analysis of various diseases, providing a reference for further research on disease prevention and treatment [20, 21]. Therefore, this study intends to apply bibliometrics to analyze the relevant information of the articles on “diabetes and osteoporosis” published between January 2011 and December 2022 to improve understanding of the research history and status of current knowledge in this field, straighten out the publication trend, and explore the research highlights.

## 2 MATERIALS AND METHODS

### 2.1 Data Sources

The Science Citation Index Expanded (SCI-E) database in the Web of Science Core Collection (WoSCC) created by the American Institute of Scientific Information in 1957 was used as the main data source for retrieving works of literature in bibliometric analysis. More than 8,000 articles and citations from important journals were included in WoSCC, which provides a comparative and standardized set of data for export and is widely used in academia [22]. SCI-E, as a valuable citation retrieval tool, is widely used in bibliometrics and scientific research evaluation. In view of the above, we select SCI-E as the target database for retrieval and analysis of this study.

### 2.2 Search Strategy

The literature retrieval was independently completed by two investigators. The retrieved publications had to satisfy the following criteria:

1. The search terms were determined by the TS (“diabetes” OR “diabetes Mellitus”) AND TS= (“osteoporosis”) ;
2. The language of publication was English, and the document types were “article” and “reviews” ;
3. The publication period was between 2011 and 2022;
4. The following information was collected: publications, authors, countries, institutions, journals, keywords, and citations.

#### 2.2.1 Inclusion and Exclusion

A total of 3,372 literatures were selected for further bibliometric analysis. Reports were excluded based on the following parameters:

1. Editorial materials (n=32)
2. Proceeding papers (n=13)
3. Letters (n=5)
4. Corrections (n=4)
5. Retraction (n=1)

### 2.3 Data Collection

All the records conforming to inclusion criteria were exported in plain-text format with full records and references, and key contents were extracted from the works of literature, such as article title, author, publication year, abstract, keywords, and citation frequency. Next, we used Microsoft Excel (Version 2019; Microsoft Corporation; Washington, United States) [23] and CiteSpace software (Version V; Drexel University; Pennsylvania, United States) [24] to perform data statistics and visual analysis.

### 2.4 Statistics and Analysis

The purpose of this study was to capture and describe the research status and future development trends of diabetes and osteoporosis. No comparison between groups or correlation analysis was performed, but citespace software was used for bibliometrics to analyze the countries/regions, institutions, authors, keywords, and journals in the articles, as well as the citation analysis and collaborative relationship visualization network rendering.

## 3 RESULTS

### 3.1 General Characteristics of the Retrieved Documents

**Fig. (1):**
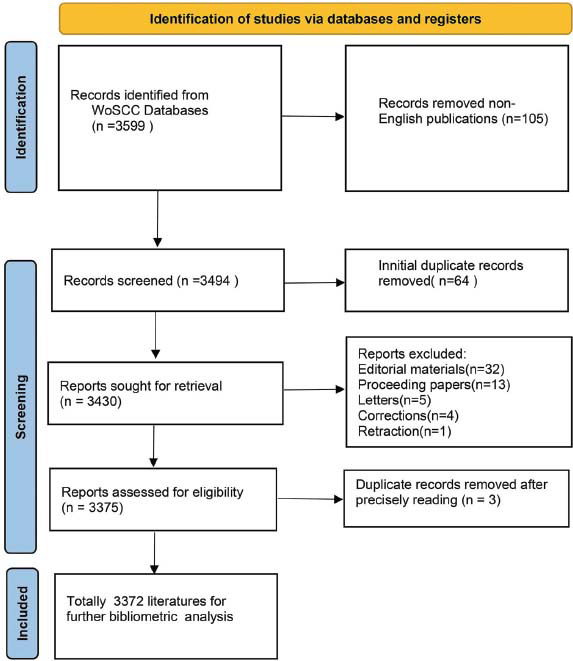
Retrieval process flowchart for the research.

**Fig. (2):**
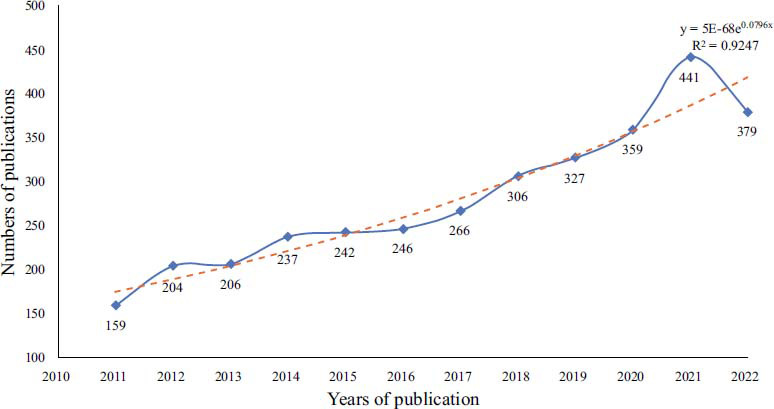
Annual trend in the number of publications.

**Fig. (3):**
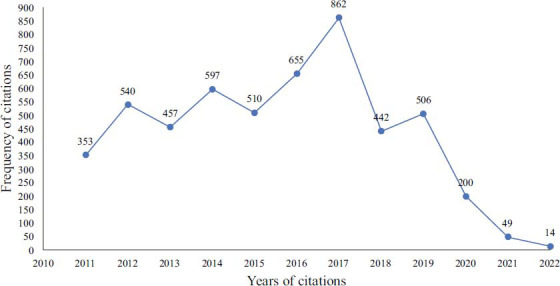
Annual trend in citations.

A total of 3599 documents from the previous decade were initially retrieved from SCI-E in WoSCC, 105 non-English publications were excluded, and 3494 articles remained, including 2560 articles, 815 review articles, 78 meeting abstracts, 32 early access, 32 editorial materials, 20 book chapters, 13 proceeding papers, 5 letters, 4 corrections, and 1 retraction; however, it should be noted that 64 of the above classifications were duplicate. Thus, the total number of articles was 3560. Finally, 3375 papers and reviews were included, and only 3372 documents could be used for further analysis after removing 3 duplicates (Fig. **1**). In terms of annual change, the number of yearly publications increased from 159 in 2011 to a peak of 441 in 2021, which showed a gradual growth trend. The polynomial fitting curve of annual publications showed an upward trend, with the correlation coefficient R2 = 0.9452 (Fig. **2**). The number of citations showed a significant increase year by year before 2017 and then decreased conspicuously to the lowest value in 2022 (Fig. **3**).

### 3.2 Country and Academic Institution Distribution

**Fig. (5):**
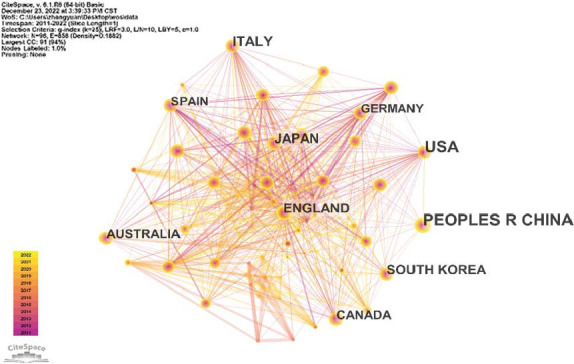
Country visualization map.

**Fig. (6):**
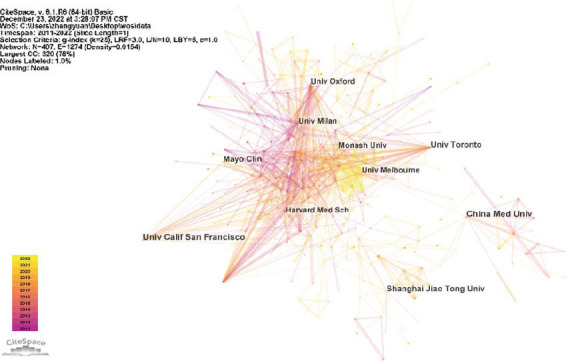
Institutional visualization map.

Research on diabetes and osteoporosis will be conducted in 96 different nations. The geographical distribution of publications is shown in Fig. (**4**). The numbers show that from 2011 to 2022, the USA has published 790 articles, ranking first in the number of published articles, followed by China, Italy, England, and Japan, with 690, 256, 230, and 212, respectively. To further explore the cooperation relationship between countries, CiteSpace software was used to perform and generate a visualization map of countries (Fig. **5**). It can be seen that 96 nodes and the 858 connections between two nodes were listed on the visualization map of countries; that is, 96 countries were listed, and 858 collaborations were made between any two countries. In addition, a similar map was produced for the organization (Fig. **6**). The number of network nodes was 407, and the number of connections was 1274. In other words, a total of 407 research institutions participated in the research in this field, including 1274 cooperations between institutions.

The statistical results show that the top 5 countries in terms of the number of publications are the United States, China, Italy, England, and Japan (Table **1**). Centrality is an index that indirectly reflects the cooperation between a country and other countries. The top five countries in cooperation are the United States, Italy, England, Brazil, and Australia (Table **2**). The top five institutions in the number of publications are the University of California-San Francisco, China Medical University, University of Toronto, Shanghai Jiao Tong University, and Mayo Clinic (Table **3**). In descending order of centrality, the top five were the University of Melbourne, China Medical University, University of California-San Francisco, University of Milan, and Harvard Medical School, all of which were greater than 0.10 (Table **4**).

### 3.3 Journal Distribution

The 3372 articles in this field were from 1161 academic journals, Among which only 10 journals published more than 30 articles, amounting to a total of 574 articles and accounting for 17% of the total works of literature (Table **5**). According to these results, it was not difficult to find that journals, such as Osteoporosis International, Plos One, and Bone have contributed considerable influence compared with others. This kind of journal mainly includes works of literature related to bone metabolism, mineral metabolism, or endocrinology. At the same time, we analyzed the citations of journals and found that the top 5 journals were Journal of Bone and Mineral Research, Osteoporosis International, Journal of Clinical Endocrinology & Metabolism, and Bone and Diabetes Care (Table **6**). The top 5 journals for centrality were Cancer Epidemiology Biomarkers & Prevention, Brain Research, Journal of Immunology, Journal of Agricultural and Food Chemistry, and Journal of Orthopaedic Surgery and Research (Table **7**).

### 3.4 Author Distribution

**Fig. (7):**
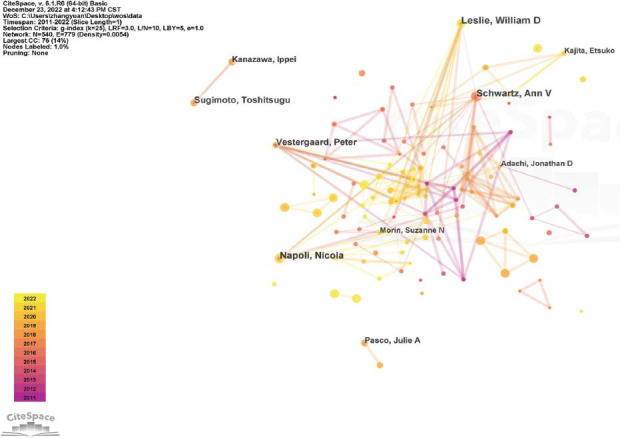
Author collaboration visualization map.

Citespace software was used for visual analysis of authors and cooperation between authors. It was found that the authors with a high number of publications included William D, Ann V, Nicola, Peter, and Toshitsugu (Table **8**), and just 7 authors had a centrality score above 0.01 (Table **9**). The map suggested that there is relatively little cooperation between authors, which was in accordance with the low centrality scores of the authors (Fig. **7**).

**Fig. (8):**
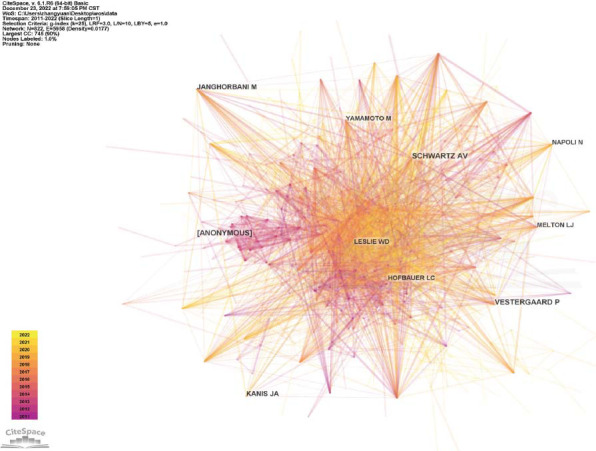
Author co-citation visualization map.

The same method should be applied to the analysis of cited authors, and the top 5 most-cited authors were Vestergaard P, Anonymous, Schwartz AV, Kanis JA, and Janghorbani M (Table **10**), and the top 5 most-cited authors ranking with centrality score were Lecka-Czernik B, Thrailkill KM, Eller-Vainicher C, Yamamoto M and Kanazawa I (Table **11**). As the author's co-citation visualization map shows, there was a wide range of links between citations, which indirectly reflected the close relationship in the same field (Fig. **8**).

### 3.5 Keywords Analysis

**Fig. (9):**
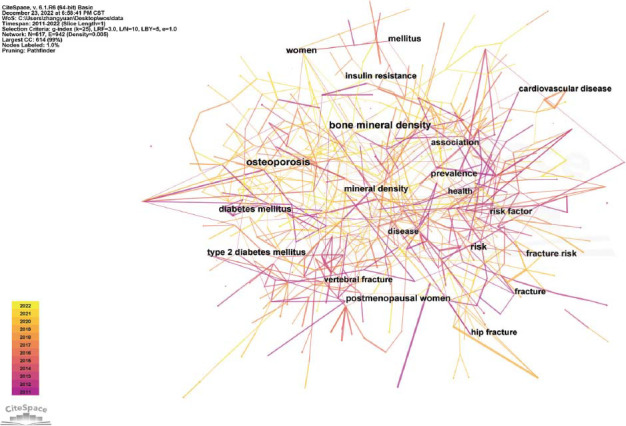
Keyword co-occurrence diagram.

**Fig. (10):**
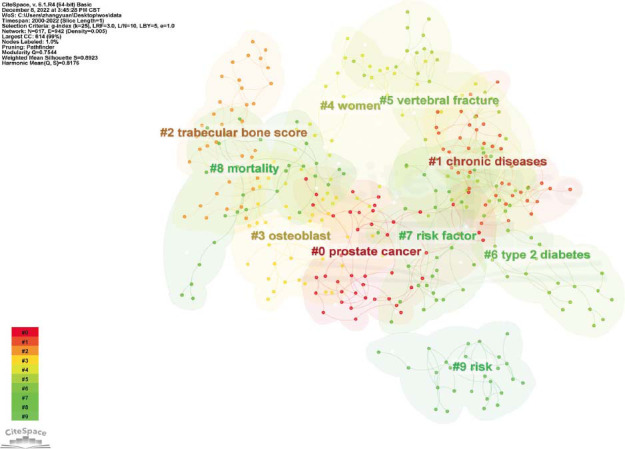
Cluster graph of keywords.

**Fig. (11):**
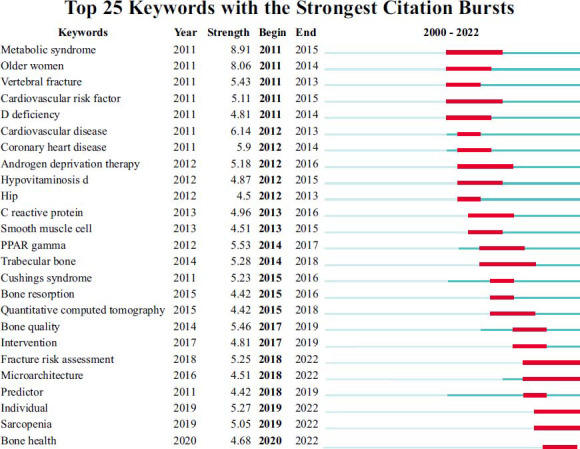
Top 25 keywords with the strongest citation bursts.

**Fig. (12):**
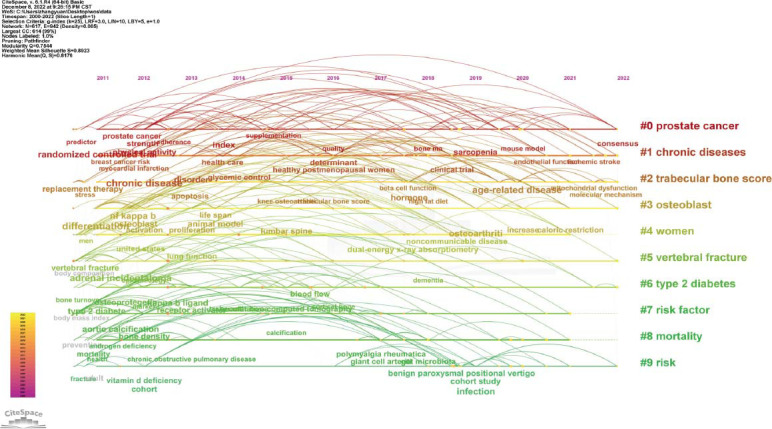
Timeline cluster graph of keywords.

Keywords co-occurrence analysis visually displayed hotspots related to diabetes and osteoporosis by studying high-frequency keywords. In this study, we used CiteSpace software to analyze the use of keywords and generate a keyword co-occurrence map. There were 617 keywords and 942 links between them (Fig. **9**). The top 5 most frequently used keywords were “osteoporosis”, “ bone mineral density”, “risk”, “diabetes mellitus”, and “postmenopausal women” (Table **12**). The top 5 keywords of centrality were “Breast cancer”, “Adipose tissue”, “Bone formation”, “Age” and “Parathyroid hormone” (Table **13**). CiteSpace was also used to conduct keyword cluster analysis (Fig. **10**), burst analysis of the keywords with a high frequency (Figs. **11** and **12**), and timeline distribution of keyword cluster analysis (Fig. **12**). The results showed that the use of hot keywords changed over time.

## 4 DISCUSSION

Diabetes is a common and chronic endocrine disease prevalent in all regions of the globe. In recent years, more and more studies have shown that type 1 and type 2 diabetes are closely related to the increased prevalence of osteoporosis. As a result, a new term, “diabetes osteoporosis (DOP)” appears, which is recognized and accepted by more and more scholars as a chronic complication of diabetes. It is reported that osteoporosis is 4-5 times higher in people with DM compared to non-diabetic people, which is also the fundamental reason why diabetes patients are prone to fractures in their lives [25]. The potential pathogenesis between diabetes and osteoporosis is intricate and complicated. In addition to chronic hyperglycemia, glycosylation end products, estrogen levels, oxidative stress, and other factors have also been widely studied. Although the related research is in full swing, there is no good analysis of the current and future research hotspots. In this work, we conducted a bibliometric analysis of publications with the theme of “diabetes and osteoporosis” to master the current status and trends of global research.

The search time was limited to 2011 - 2022, and 3372 articles were finally included for bibliometric analysis. We comprehensively and systematically analyzed the distribution of countries/institutions/journals/authors/keywords in this field and summarized current research hotspots and development trends by analyzing keywords and highly cited articles. The results showed that there were a few articles related to this field before 2011, but since 2011, the publications about diabetes and osteoporosis have increased significantly, and the frequency of citations has also shown a rapid growth trend. The United States and China had the most publications on this topic. University of California San Francisco and China Medical University published the largest number of documents, and the University of Melbourne cooperated more frequently with other institutions. Osteoporosis International was the journal with the overwhelming number of papers in this field, and the Journal of Bone and Mineral Research had been cited the most times. William D and Ann V were the authors who published the most articles in the research of diabetes and osteoporosis. Keyword analysis showed that osteoporosis, diabetes mellitus, bone mineral density, risk, and postmenopausal women were frequently used words. Among them, fracture risk assessment, bone microstructure, sarcopenia, and bone health are the hotspots of recent concern.

With regard to the contributions of countries and organizations, the United States and China, as the top two countries in the number of relevant research articles, occupy an important position in the field of diabetes and osteoporosis research. The United States, as the country with the highest health expenditure on diabetes in the world, combined with the aging population, had to force medical and health institutions and science and technology departments to study this kind of chronic disease and seek new interventions. The United States had unique advantages in basic and clinical medical research, including adequate funds, advanced equipment and professional researchers. Among its outstanding institutions, such as the University of California San Francisco, Mayo Clinic, and Harvard Medical School, many high- level research achievements have been published, promoting the global comprehensive and multi-level sharing of scientific research achievements to a certain extent. As a country with the largest number of diabetes patients and the most severe trend of population aging in the world, China was making every effort to carry out joint research on chronic diseases, such as diabetes and osteoporosis, and institutions, such as China Medical University and Shanghai Jiaotong University have made unprecedented progress in this field. It has special practical significance to improve the quality of life of patients.

Research achievements in this field have been published in many medical journals, such as Osteoporosis International, Plos One, Bone, *etc.* More than half of the top ten journals are related to osteoporosis, bone metabolism, and endocrinology. The results of literature citation analysis show that the Journal of Bone and Mineral Research is the most cited journal, and the New England Journal of Medicine, JAMA Journal of the American Medical Association, and Lancet, as top journals, are cited far more frequently than other journals. Leslie, William D., as the most prolific author, has made great contributions to the study of diabetes and osteoporosis. Keyword co-occurrence analysis can reflect the development trend and hot spots of related research to a certain extent and reveal the potential research value. The results of keyword analysis show that in addition to diabetes and osteoporosis, bone mineral density, postmenopausal women, and risk factors have also been research hotspots in recent years.

## CONCLUSION

At present, the relationship between diabetes and osteoporosis has been confirmed. Although the mechanism of type 1 and type 2 diabetes leading to osteoporosis is different, the core of its pathogenesis is the destruction of bone metabolism and the balance of the internal environment of the bone [26-28]. Long-term chronic hyperglycemia will reduce the synthesis and release of insulin-like growth factors, thereby inhibiting the proliferation and differentiation of osteoblasts. Starup Linde found that the synthesis and secretion of osteocalcin by osteoblasts in diabetes will be inhibited, and the process of bone mineralization will be affected, leading to the reduction of bone mineral content [29]. Nayak found that diabetes leads to insufficient bone blood supply, bone microstructure destruction, and bone density reduction. Moreover, the relative or absolute lack of insulin will affect the binding of insulin receptors on the surface of osteoblasts to insulin and ultimately reduce the synthesis of collagen and bone quality [30]. In a meta-analysis, it was mentioned that osteoporosis affects more than one-third of diabetes patients in the Chinese Mainland, of which women and the elderly are at higher risk and may need clinical prevention [31].

## LIMITATIONS 

However, some limitations of this study should be noted. First of all, the SCI-E database was the only data source for this search, which may cause some works of literature to be omitted. Secondly, the retrieval strategy was limited to topic term retrieval, which might have missed documents that could be retrieved by other retrieval. Finally, we excluded articles in languages other than English, which was also a factor leading to partial deviation in the results.

## Figures and Tables

**Fig. (4):**
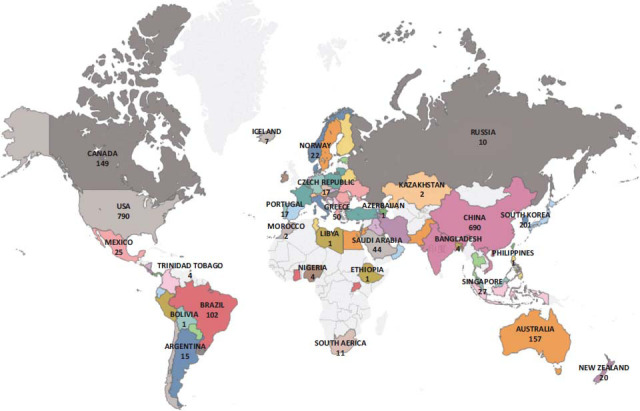
Geographical distribution of publications.

**Table 1:** Top 10 countries by number of publications.

Rank	Countries	Publications
1	USA	790
2	China	690
3	Italy	256
4	England	230
5	Japan	212
6	South korea	201
7	Australia	157
8	Canada	149
9	Germany	148
10	Spain	136

**Table 2:** Top 10 countries by centrality.

Rank	Countries	Centrality
1	USA	0.32
2	Italy	0.17
3	England	0.17
4	Brazil	0.13
5	Australia	0.07
6	India	0.07
7	Switzerland	0.07
8	Spain	0.06
9	Canada	0.05
10	Japan	0.04

**Table 3:** Top 10 institutions by number of publications.

Rank	Institutions	Publications
1	University of California-San Francisco	46
2	China Medical University	44
3	University of Toronto	40
4	Shanghai Jiao Tong University	40
5	Mayo Clinic	39
6	University of Milan	33
7	Monash University	33
8	University of Melbourne	32
9	Harvard Medical School	31
10	University of Oxford	30

**Table 4:** Top 10 institutions by centrality.

Rank	Institutions	Centrality
1	University of Melbourne	0.18
2	China Medical University	0.15
3	University of California-San Francisco	0.14
4	University of Milan	0.14
5	Harvard Medical School	0.14
6	University of Sheffield	0.11
7	University of Southampton	0.09
8	University of Oxford	0.08
9	Columbia University	0.08
10	University of Toronto	0.07

**Table 5:** Top 10 journals by number of publications.

Journal	Records	% (N=3372)
Osteoporosis International	130	3.86
Plos One	68	2.02
Bone	67	1.99
Frontiers in Endocrinology	57	1.69
Journal of Clinical Endocrinology & Metabolism	56	1.66
Journal of Bone and Mineral Research	53	1.57
Calcified Tissue International	40	1.19
Journal of Bone and Mineral Metabolism	39	1.16
Medicine	33	0.98
Nutrients	31	0.92

**Table 6:** Top 10 journals with the most cited publications.

Rank	Journal	Frequency
1	Journal of Bone and Mineral Research	1732
2	Osteoporosis International	1726
3	Journal of Clinical Endocrinology & Metabolism	1578
4	Bone	1508
5	Diabetes Care	1198
6	Plos One	1179
7	New England Journal of Medicine	1150
8	JAMA-Journal of the American Medical Association	1029
9	Lancet	1025
10	Calcified Tissue International	978

**Table 7:** Top 10 journals cited by centrality.

Rank	Journal	Centrality
1	Cancer Epidemiology Biomarkers & Prevention	0.05
2	Brain Research	0.04
3	Journal of Immunology	0.03
4	Journal of Agricultural and Food Chemistry	0.03
5	Journal of Orthopaedic Surgery and Research	0.03
6	Clinical Science	0.03
7	Osteoporosis International	0.02
8	Journal of Biological Chemistry	0.02
9	Proceedings of the National Academy of Sciences of the United States of America	0.02
10	Nature	0.02

**Table 8:** Top 10 authors by number of publications.

Rank	Authors	Publications
1	Leslie, William D	15
2	Schwartz, Ann V	14
3	Napoli, Nicola	13
4	Vestergaard, Peter	11
5	Sugimoto, Toshitsugu	11
6	Kanazawa, Ippei	9
7	Pasco, Julie A	9
8	Adachi, Jonathan D	7
9	Morin, Suzanne N	7
10	Kajita, Etsuko	7

**Table 9:** Top-ranked authors by centrality.

Rank	Authors	Centrality
1	Leslie, William D	0.02
2	Schwartz, Ann V	0.02
3	Napoli, Nicola	0.01
4	Rosen, Clifford J	0.01
5	Cooper, Cyrus	0.01
6	Bouxsein, Mary L	0.01
7	Kanis, John A	0.01

**Table 10:** Top 10 authors by citations.

Rank	Authors	Frequency
1	Vestergaard P	513
2	Anonymous	464
3	Schwartz AV	434
4	Kanis JA	358
5	Janghorbani M	316
6	Leslie WD	239
7	Napoli N	236
8	Hofbauer LC	219
9	Yamamoto M	205
10	Melton LJ	191

**Table 11:** Top 10 cited authors by centrality.

Rank	Authors	Centrality
1	Lecka-Czernik B	0.07
2	Thrailkill KM	0.06
3	Eller-Vainicher C	0.05
4	Yamamoto M	0.04
5	Kanazawa I	0.04
6	Saito M	0.04
7	De Liefde II	0.04
8	Looker AC	0.04
9	Loke YK	0.04
10	Bischoff-Ferrari HA	0.04

**Table 12:** Top 5 keywords by frequency.

Rank	Keywords	Frequency
1	Osteoporosis	727
2	Bone mineral density	726
3	Risk	493
4	Diabetes mellitus	396
5	Postmenopausal women	387

**Table 13:** Top 5 keywords by centrality.

Rank	Keywords	Centrality
1	Breast cancer	0.06
2	Adipose tissue	0.05
3	Bone formation	0.05
4	Age	0.04
5	Parathyroid hormone	0.04

## Data Availability

The original contributions presented in the study are included in the article. Further inquiries can be directed to the corresponding author.

## LIST OF ABBREVIATIONS

DOP: Diabetes Osteoporosis
IGF-1: Insulin-Like Growth Factor-1
SCI-E: Science Citation Index Expanded
SHBG: Sex Hormone Binding Globulin
WoSCC: Web of Science Core Collection

## STANDARDS OF REPORTING

PRISMA guidelines and methodology were followed.

## SUPPLEMENTARY MATERIAL

PRISMA checklist is available as supplementary material on the publisher’s website along with the published article.
